# Switch to aflibercept or ranibizumab after initial treatment with bevacizumab in eyes with neovascular AMD

**DOI:** 10.1186/s12886-017-0471-x

**Published:** 2017-05-23

**Authors:** Maria Waizel, Margarita G. Todorova, Michael Masyk, Katharina Wolf, Annekatrin Rickmann, Khaled Helaiwa, Björn R. Blanke, Peter Szurman

**Affiliations:** 1 Department of Ophthalmology, University of Basel, Mittlere Strasse 91, CH-4031 Basel, Switzerland; 2 Knappschaft Eye Clinic Sulzbach, Knappschaft Hospital Saar, Sulzbach/Saar, Germany; 3 University Eye Clinic Tuebingen, Centre for Ophthalmology, Tuebingen, Germany

**Keywords:** Vascular endothelial growth factor a, Ranibizumab, Aflibercept, Bevacizumab, VEGF switch, Vascular endothelial growth factor

## Abstract

**Background:**

To evaluate changes in central macular thickness (CMT) and visual outcome in patients with neovascular age-related macular degeneration (AMD) treated initially with bevacizumab and subsequently switched to either aflibercept or ranibizumab.

**Methods:**

Observational clinical study was performed. We measured the structural outcome (CMT on SD-OCT; μm) and the visual outcome (best corrected visual acuity (BCVA); logMAR), as follows: before treatment (at baseline), following bevacizumab treatment (switch follow-up) and after switching from bevacizumab to aflibercept- or ranibizumab treatment (final follow-up, AG/, RG).

**Results:**

From a total of 96 eyes treated with intravitreal injections of bevacizumab (10.5 ± 7.6 (mean ± SD)), 58 eyes switched to aflibercept (6.5 ± 3.9; AG) and 38 eyes switched to ranibizumab (7.1 ± 5.3; RG) (≥ 3 injections, each). In addition, these eyes were compared to 37 eyes under bevacizumab monotherapy.

Primary outcome: In the AG, the CMT decreased slightly from 430 ± 220 μm at baseline to 419 ± 212 μm at switch follow-up (*p* = 0.86), but decreased significantly to 318 ± 159 μm at final follow-up, AG (*p* < 0.0001). In the ranibizumab group (RG), the CMT increased from 396 ± 174 μm at baseline to 499 ± 333 μm at switch follow-up (*p* = 0.012), but decreased significantly to 394 ± 202 μm at final follow-up, RG (*p* = 0.007).

Secondary outcome: In the AG, the mean BCVA worsened from logMAR 0.57 ± 0.33 at baseline to 0.63 ± 0.30 at switch follow-up and improved slightly to 0.53 ± 0.71 at final follow-up, AG (*p* = 0.46). In the RG, mean BCVA worsened from 0.57 ± 0.28 at baseline to 0.64 ± 0.31 at switch follow-up and improved slightly to 0.60 ± 0.36 at final follow-up, RG (*p* = 0.64).

**Conclusion:**

Switching from bevacizumab to either aflibercept, or ranibizumab, has a strong anatomical effect in eyes with neovascular AMD. Nevertheless, even if the switch to aflibercept shows a minimal functional benefit over that to ranibizumab, visual prognosis remains limited.

## Background

Intravitreal injections of vascular endothelial growth factor inhibitors (anti-VEGF) are the gold standard for treatment in eyes with exudative age-related macular degeneration (AMD). However, long-term monotherapy with anti-VEGF often leads to reduced effectiveness over time. So far, the underlying pathophysiology is not well understood and ranges from tolerance to tachyphylaxis, e.g. by up-regulation of alternative pathways or autoantibodies [1, 2].

Various therapeutic strategies have been proposed to prevent visual loss progression during the long-term use of anti-VEGF. For instance, switching anti-VEGF drugs over time seems to reduce the rate of “poor” responders to anti-VEGF monotherapy [3–5]. Many patients show medical histories with multiple drug switches, since the variety of anti-VEGF agents presented on the market offers multiple combinations. However, the interactions after these switches are not well understood.

Aflibercept (Eylea®, VEGF Trap-Eye, Bayer HeathCare) was assumed to have a higher binding affinity for VEGF-A than ranibizumab (Lucentis®, Novartis AG) and bevacizumab (Avastin®, F. Hoffmann-La Roche AG) [6]. Contrariwise, a recent study demonstrated a distinct superior VEGF-binding affinity of ranibizumab over aflibercept [7]. Thus, this issue remains to be elucidated. Especially the question of the clinical impact of these observations is still vague. Several recent publications of mainly small, retrospective case series focused on the switch from ranibizumab and/or bevacizumab to aflibercept [3, 8–14]. These investigations showed a significant anatomical effect with a decrease in central macular thickness. Nevertheless, the switch to aflibercept resulted in a poor visual outcome with no significant benefit in regard to BCVA [3, 9, 11, 12, 14].

Switching from bevacizumab to ranibizumab has already demonstrated a strong anatomical, but limited functional benefit [5, 15, 16]. However, no investigation has been performed to ascertain whether the effect of ranibizumab and aflibercept is comparable. If aflibercept is assumed to have a higher affinity to VEGF, aflibercept could be expected to be more effective and thus, to have a better functional and anatomical outcome, than ranibizumab.

To our knowledge, no systematic comparison on the functional and anatomical outcome of aflibercept versus ranibizumab treatment after initial bevacizumab regimen, has been perfomed so far. Thus, the aim of this study was to investigate, under real-life conditions in a single vitreoretinal center, the outcome of intravitreal aflibercept versus ranibizumab treatment after initial bevacizumab regimen. Furthermore, in order to find a reasonable therapeutic strategy for future clinical decisions, the second purpose of this study, was to verify whether a switch from bevacizumab to aflibercept or to ranibizumab is associated with better outcome.

## Methods

Observational clinical study on 133 eyes of which 96 eyes of 74 consecutive AMD patients were treated with intravitreal injections of aflibercept (aflibercept group (AG)), or ranibizumab (ranibizumab group (RG)), after initial regimen with intravitreal bevacizumab, was performed and compared to a control group with 37 eyes of 33 consecutive AMD patients under bevacizumab monotherapy. We collected the data from March 2011 until April 2017 in a single vitreoretinal surgery center (Knappschaft Eye Clinic Sulzbach). This study was approved by the local authorities (Ethics Commission of the State Chamber of Medicine in Saarland, trial number 73/15) with a positive vote for prospective observational investigations from April 2015 onwards.

Patients who met the following criteria were included in this study:

- vision impairment due to neovascular AMD,
- no previous anti-VEGF treatment before initial intravitreal injection of bevacizumab,
- at least three monthly applied intravitreal injections of 2 mg aflibercept (Eylea®, VEGF Trap-Eye, Bayer HeathCare) or 0.5 mg ranibizumab (Lucentis®, Novartis AG) after initial treatment with at least three monthly applied intravitreal injections of 1.25 mg bevacizumab (Avastin®, F. Hoffmann-La Roche AG) for AG and RG,
- available examination data at the last visit before treatment initiation (“baseline”), at 4 weeks after the last intravitreal injection of bevacizumab (“switch follow-up” visit) and at the last follow-up visit 4 weeks after switching from bevacizumab to aflibercept- or ranibizumab treatment (“final follow-up” visit, AG or RG).
- control group: The last available visit 4 weeks after last injection of bevacizumab monotherapy was taken into account as “final follow-up” and compared to AG and RG.

Following our standardized clinical protocol all our patients received a serial of three monthly anti-VEGF injections, followed by a visit 4 weeks after the last injection. Supplementary, since the VIEW-study recommended to dose aflibercept every 2 months after 3 initial monthly doses [17], we also included all available results of an optional 8 weeks follow-up, which was attended by 64% of the AG and by 66% of the RG.

Switching to aflibercept or ranibizumab regimen was either due to vision impairment, caused by persisting or increasing sub- or intraretinal fluid after previous anti-VEGF treatment, as verified by spectral domain optical coherence tomography (SD-OCT), or internal non-medical policy decisions.

Patients were excluded from this study, if they:

- did not receive a treatment with at least three intravitreal injections of bevacizumab, ranibizumab or aflibercept, respectively,
- or did not attend the baseline, the switch or the final follow-up visit.
- Eyes with previous anti-VEGF treatment before the initial intravitreal injection of bevacizumab (1.25 mg), were excluded from the study.

Primary study endpoint was central macular thickness (CMT, μm) determined by SD-OCT, secondary study endpoint was best corrected visual acuity (BCVA). At baseline, at switch- and at final follow-up visit, a slit lamp examination of the retina and anterior segment, applanation tonometry and BCVA were performed. Switch follow-up and final follow-up visits were performed 4 weeks after the last injection. BCVA was converted to logMAR for statistical analysis. Central macular thickness (CMT) was measured via the software’s ruler tool in μm on the OCT image and was measured from the internal limiting membrane to the retinal pigment epithelium with Spectralis ® OCT (Heidelberg Engineering, Heidelberg Germany, Software Version 1.8.6.0).

For statistical evaluation non-parametric tests (Friedman test for multiple dependent comparisons, Kruskall-Wallis test for multiple independent comparisons, paired Wilcoxon rank sum test for dependent pairwise comparisons and unpaired Mann Whitney U-Test for independent pairwise comparisons between different groups) with Matlab® R2014b (The MathWorks, Version 8.4.0), were performed. *P* < 0.05 was defined as statistically significant.

## Results

A total of 133 eyes were included in the study. 96 eyes of 74 patients from AG or RG were compared to a control group consisting of 37 eyes of 33 consecutive AMD patients under bevacizumab monotherapy. The median age (± SD; range) of our switch patients was 76.7 (± 9.0; 52–94) years; 45 patients were women and 29 were men. The median age (± SD; range) of our controls was 79.1 (± 6.7; 62–96) years; 21 patients were women and 12 were men.

**At switch follow-up**, a total of 10.5 ± 7.6 (mean; ± SD; range 3–33) intravitreal injections of bevacizumab were applied prior to aflibercept or ranibizumab switch (in AG: 13.2 ± 8.3; range: 3–33 and in RG: 6.3 ± 3.3; range: 3–18, respectively). The mean switch follow-up visit (± SD) was performed 5.4 weeks (± 3.3) after last bevacizumab injection (in AG: 5.0 weeks (± 1.4) and in RG: 6.1 weeks (± 4.8), respectively). The mean duration of bevacizumab treatment (± SD) before switching to AG or RG was 11.6 weeks (± 6.7) (in AG: 11.9 weeks (± 6.3) and in RG: 10.9 weeks (± 7.3), respectively; *p* = 0.16).

**Final follow up**: 58 eyes were switched to 6.5 ± 3.9 (range 3–16) injections of aflibercept in the AG and 38 eyes to 7.1 ± 5.3 (range 3–23) injections of ranibizumab In the RG. The final follow-up visit was performed 4.8 weeks (± 1.2) after last injection in the AG and 4.5 weeks (± 1.4) in the RG. In the 37 eyes of the control group, the final follow-up visit was performed after 17.2 ± 6.2 injections (range 6–27) with a mean duration of 11.2 ± 5.2 weeks of bevacizumab monotherapy. Since the VIEW-study recommended to dose aflibercept every 2 months after 3 initial monthly doses, we also examined all available results at supplemetary follow-up visit 8 weeks after the last treatment, attended by 64% (37 out of 58 eyes) in the AG and 66% (25 out of 38 eyes) in the RG.

### Primary study endpoint - reduction of mean CMT- both groups (Fig. 1)

In the AG, CMT decreased slightly from 430 ± 220 μm at baseline to 419 ± 212 μm at switch follow-up visit (*p* = 0.86, Wilcoxon pairwise comparison) and decreased significantly to 318 ± 159 μm at final follow-up visit, AG (*p* < 0.0001). At the supplementary 8 weeks follow-up, CMT remained stable with 315 ± 222 μm (*p* = 0.06).

**Fig. 1:**
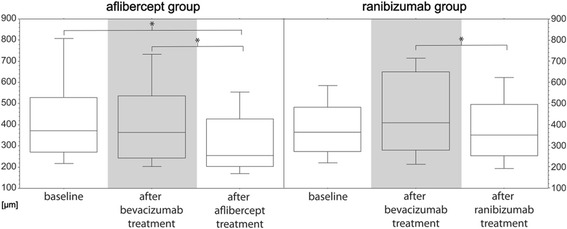
*Box Plot* analysis illustrates central macular thickness in μm in eyes prior to treatment, at switch follow-up visit after treatment with bevacizumab (*grey background*) and at final follow-up visit after treatment with aflibercept (*left side*) and after treatment with ranibizumab (*right side*). The ordinate shows central macular thickness in μm for eyes at baseline visit prior to treatment (*left box*), at switch follow-up visit after treatment with bevacizumab (*middle*) and at final follow-up visit after treatment with aflibercept or ranibizumab (*right box*) shown on the abscissa. Statistically significant results (pairwise comparison Wilcoxon test, *p* < 0.05) are marked with an asterisk. Note that for AG there was a statistically significant reduction of mean central macular thickness compared at baseline and after aflibercept treatment (*p* = 0.0001) whereas for RG there was no statistically significant difference between baseline and final follow-up visit (*p* = 0.67)

In the RG, CMT increased from 396 ± 174 μm at baseline to 499 ± 333 μm at switch follow-up visit (*p* = 0.012) and decreased significantly to 394 ± 202 μm at final follow-up visit, RG (*p* = 0.007). At the supplementary 8 weeks follow-up, CMT decreased slightly to 326 ± 164 μm (*p* = 0.88).

When the CMT difference between the final follow-up visit and the baseline was taken into account, the AG showed a significant reduction from 430 ± 220 μm at baseline to 318 ± 159 μm at final follow-up visit (*p* = 0.0001). However, this was not the case for the RG (*p* = 0.67).

In addition, regarding the CMT at the supplementary 8 weeks follow-up, we found a statistically significant reduction for AG, when compared to baseline (*p* = 0.002) and to switch follow-up (*p* = 0.03), whereas for RG this was again not the case (*p* = 0.59 and *p* = 0.58, respectively).

Figure 1 illustrates the results as a boxplot analysis. Since the supplementary follow-up 8 weeks after treatment was optional and, therefore was not attended by all of the patients, it is not included in the Figure. Statistically significant results of pairwise comparisons (*p* < 0.05) are marked with an asterisk.

### Secondary study endpoint - improvement of BCVA - both groups

In the AG, mean BCVA ± SD decreased from logMAR 0.57 ± 0.33 at baseline to logMAR 0.63 ± 0.30 at switch follow-up, and increased slightly to logMAR 0.53 ± 0.71 at final follow-up, AG (*p* = 0.46). In the RG, mean BCVA decreased from logMAR 0.57 ± 0.28 at baseline to logMAR 0.64 ± 0.31 at switch follow-up, and increased slightly to logMAR 0.60 ± 0.36 at final follow-up, RG (*p* = 0.64, Friedman test, Table 1).

**Table 1:**
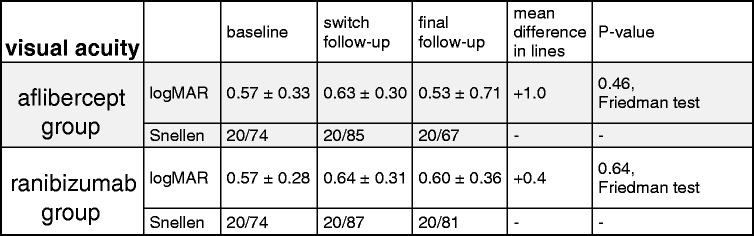
Table data illustrates visual acuity at baseline visit prior to treatment, at switch follow-up visit after treatment with bevacizumab and at final follow-up visit after treatment with aflibercept (grey background) and after treatment with ranibizumab (white background)

In both groups, there was no statistically significant difference for pairwise comparisons between the baseline, the switch- and the final follow-up visit. Nevertheless, at final follow-up an overall gain in BCVA of 1.0 line was achieved in AG and of 0.4 lines in RG. At the supplementary 8 weeks follow-up, the mean BCVA decreased slightly to logMAR 0.60 ± 0.35 μm (*p* = 0.95) in AG, but remained stable at logMAR 0.59 ± 0.34 μm (*p* = 0.81) in RG .

To rule out a possible bias of non-homogeneous group formation before switching to either ranibizumab or aflibercept we calculated the inter-group characteristics at baseline, at switch follow-up, at final follow-up and at supplemetary follow-up (8 weeks after the last treatment). There was neither a statistically significant difference between the groups at baseline (*p* = 0.95) nor at switch follow-up (*p* = 0.82), final follow-up (*p* = 0.65) nor at the supplementary 8 weeks follow-up (*p* = 0.84). Similar results could be shown for mean CMT within both groups. Again there was neither a statistically significant difference between the groups at baseline (*p* = 0.42) nor at switch follow-up (*p* = 0.60), final follow-up (*p* = 0.18) or at the supplementary 8 weeks follow-up (*p* = 0.50).

### Comparison of both groups to controls

In the control group, CMT decreased slightly from 387 ± 148 μm at baseline to 351 ± 144 μm at final follow-up visit (*p* = 0.16, Wilcoxon pairwise comparison), whereas the mean BCVA ± SD remained stable with logMAR 0.68 ± 0.28 at baseline and logMAR 0.66 ± 0.26 at final follow-up (*p* = 0.60).

When all three subgroups (AG, RG and controls) were compared at baseline and at final follow-up visit, we found neither a significant difference for BCVA both, at baseline (*p* = 0.10, Kruskall Wallis test), and at final follow-up (*p* = 0.59), nor for the CMT (*p* = 0.94 and *p* = 0.10, respectively). All subsequent pairwise comparisons presented with no significant difference between the AG, RG and controls (*p* > 0.05).

In nine eyes postoperative complications occurred. Three eyes were highly suspicious for an endophthalmitis after injection and were treated with vitrectomy and intravitreal application of antibiotics, four eyes were highly suspicious for retinal pigment epithelial tear, one eye (AG) showed a submacular hemorrhage and was treated with vitrectomy and subretinal application of recombinant tissue plasminogen activator (rtPA). After occurrence of this hemorrhage the patient was excluded from further investigation.

## Discussion

To the best of our knowledge, this is the first study that compared the efficacy of aflibercept versus ranibizumab treatment after initial bevacizumab regimen. Several recent publications of mainly small, retrospective case series focused predominantly either on the switch to aflibercept for eyes non-responding to ranibizumab and/or bevacizumab, favouring the switch to aflibercept [3, 8–14]. Some other studies focused on the switch from bevacizumab to ranibizumab only [5, 15, 16]. Nevertheless, it remains unclear whether a treatment with aflibercept is more effective e.g. due to a higher affinity to VEGF or possible cross tolerance effects between ranibizumab and bevacizumab. In addition, the question whether a switch of anti-VEGF drugs over time can also be a treatment option, has not finally been answered yet.

Our results are reflecting clinical reality outside randomized trials and confirm the efficacy of treatment with both aflibercept and ranibizumab after initial bevacizumab regimen. We observed an overall gain in visual acuity of 1.0 line for switches to aflibercept and 0.4 lines for switches to ranibizumab. Although our results did not reach statistical significance, regarding the functional benefit we found aflibercept to be slightly more effective than ranibizumab.

Our results are in accordance with studies investigating both the outcome after switching from ranibizumab and/or bevacizumab to aflibercept [3, 8–14, 18] and after switching from bevacizumab to ranibizumab [5, 15, 16]. Fassnacht-Riederle et al. only found minimal functional improvement when switching to aflibercept after initial treatment with ranibizumab and/or bevacizumab: Visual acuity increased about 1.8 letters in ETDRS visual acuity score but did not reach statistical significance [3]. Bakall et al. found a statistical significant decrease in CMT in 36 eyes with exudative AMD but no significant change in visual acuity after a switch to aflibercept after initial treatment with ranibizumab and/or bevacizumab [9]. Comparable functional outcome with no statistical significant improvement of visual acuity was found for switches from bevacizumab to ranibizumab by Moisseiev et al. [15]. Therefore, our observations are in accordance with these studies.

Comparison of anatomical results revealed that both switches from bevacizumab to aflibercept and to ranibizumab were accompanied by a statistical significant decrease in central macular thickness. Meanwhile, comparing the CMT at baseline to final follow-up visit we found a statistically significant reduction only for aflibercept (*p* = 0.0001), but not for ranibizumab (*p* = 0.67). The same effect could be shown at the supplementary 8 weeks follow-up: We found a statistically significant reduction for aflibercept compared to baseline (*p* = 0.002) and switch follow-up (*p* = 0.03), whereas for ranibizumab there was no statistically significant reduction compared to baseline (*p* = 0.59) or switch follow-up (*p* = 0.58). Our results are in accordance with studies investigating the anatomical outcome both after switching from ranibizumab and/or bevacizumab to aflibercept [3, 8–14] and after switching from bevacizumab to ranibizumab [5, 15, 16]. Bakall et al. found a statistical significant decrease in CMT after a switch from ranibizumab and/or bevacizumab to aflibercept [9]. Hall et al. found similar functional effects after switching to aflibercept in 30 eyes with neovascular AMD with minor functional improvement [11]. Ehlken et al. showed a statistically significant reduction of CMT after switching from bevacizumab to ranibizumab [5]. Therefore, our observations show comparable results for a switch either from bevacizumab to aflibercept or to ranibizumab. In summary, we identified a clinical benefit for both treatment options with ranibizumab and aflibercept after initlal bevacizumab regimen, with a minimal advantage for aflibercept over ranibizumab. a.

So far, this is the first systematical analysis in literature that compared the efficacy of switches to aflibercept versus ranibizumab after initial bevacizumab regimen.

In conclusion, our results indicate that treatment with both aflibercept or ranibizumab after initial bevacizumab regimen can lead to significant decrease in central macular thickness and slight vision improvement. We found only a slight advantage for aflibercept over ranibizumab that should not be overinterpreted. However, it seems that drug exchange in any direction (either from bevacizumab to aflibercept or to ranibizumab) plays an important role in optimizing treatment success after long term anti-VEGF treatment. Therefore, our results suggest that careful drug selection might improve treatment outcome.

Limitations of our study may include the inhomogenous group size of observed eyes, the short follow-up time and due to the observational character of this investigation missing data about the timepoint of the first diagnosis of AMD. Nevertheless, even with a smaller number of observations in the ranibizumab group a clear anatomical benefit could be detected. Compared to other published retrospective case series that also did not mention the timepoint of the first diagnosis of exudative AMD we present data of large cohorts. In addition, since our center follows a standardized protocol with a serial of three montly anti-VEGF injections followed by a visit 4 weeks after last injection our results are more eligible for comparisons between different substances than studies with individualized treatment intervals. Further improvement, especially with regard to visual acuity, might be expected within a longer follow-up, because further treatment and rehabilitation of anatomical structures in the macular region might take place and lead to improved functional outcome.

However, we present data of the first systematic investigation on the functional and anatomical outcome of aflibercept or ranibizumab treatment after initial bevacizumab regimen. Our results demonstrated that both treatment options lead to a significant decrease in central macular thickness and slight vision improvement with a questionable advantage for aflibercept over ranibizumab. In order to optimize treatment decision further prospective studies with homogenous group size and a longer follow-up period are needed to identify baseline characteristics more precisely that are correlated with higher efficacy rates.

## Conclusion

Switching from bevacizumab to either aflibercept, or ranibizumab, has a strong anatomical effect in eyes with neovascular AMD. Nevertheless, even if the switch to aflibercept shows a minimal functional benefit over that to ranibizumab, visual prognosis remains limited.

## Abbrevations

AG: aflibercept group
AMD: age-related macular degeneration
BCVA: best corrected visual acuity
CMT: central macular thickness
RG: ranibizumab group
SD-OCT: spectral domain optical coherence tomography

## References

[1] Binder S (2012). Loss of reactivity in intravitreal anti-VEGF therapy: tachyphylaxis or tolerance?. Br J Ophthalmol.

[2] Forooghian F, Chew EY, Meyerle CB, Cukras C, Wong WT (2011). Investigation of the role of neutralizing antibodies against bevacizumab as mediators of tachyphylaxis. Acta Ophthalmol.

[3] Fassnacht-Riederle H, Becker M, Graf N, Michels S (2014). Effect of aflibercept in insufficient responders to prior anti-VEGF therapy in neovascular AMD. Graefes Arch Clin Exp Ophthalmol.

[4] Gasperini JL, Fawzi AA, Khondkaryan A, Lam L, Chong LP, Eliott D, Walsh AC, Hwang J, Sadda SR (2012). Bevacizumab and ranibizumab tachyphylaxis in the treatment of choroidal neovascularisation. Br J Ophthalmol.

[5] Ehlken C, Jungmann S, Böhringer D, Agostini HT, Junker B, Pielen A (2014). Switch of anti-VEGF agents is an option for nonresponders in the treatment of AMD. Eye (Lond).

[6] Papadopoulos N, Martin J, Ruan Q, Rafique A, Rosconi MP, Shi E, Pyles EA, Yancopoulos GD, Stahl N, Wiegand SJ (2012). Binding and neutralization of vascular endothelial growth factor (VEGF) and related ligands by VEGF trap, ranibizumab and bevacizumab. Angiogenesis.

[7] Yang J, Wang X, Fuh G, Yu L, Wakshull E, Khosraviani M, Day ES, Demeule B, Liu J, Shire SJ, Ferrara N, Yadav S (2014). Comparison of binding characteristics and in vitro activities of three inhibitors of vascular endothelial growth factor a. Mol Pharm.

[8] Pinheiro-Costa J, Costa JM, Beato JN, Freitas-da-Costa P, Brandão E, Falcão MS, Falcão-Reis F, Carneiro ÂM (2015). Switch to Aflibercept in the treatment of Neovascular AMD: one-year results in clinical practice. Ophthalmologica.

[9] Bakall B, Folk JC, Boldt HC, Sohn EH, Stone EM, Russell SR, Mahajan VB (2013). Aflibercept therapy for exudative age-related macular degeneration resistant to bevacizumab and ranibizumab. Am J Ophthalmol.

[10] Singh RP, Srivastava S, Ehlers JP, Bedi R, Schachat AP, Kaiser PK (2014). A single-arm, investigator-initiated study of the efficacy, safety and tolerability of intravitreal aflibercept injection in subjects with exudative age-related macular degeneration, previously treated with ranibizumab or bevacizumab: 6-month interim analysis. Br J Ophthalmol.

[11] Hall LB, Zebardast N, Huang JJ, Adelman RA (2014). Aflibercept in the treatment of neovascular age-related macular degeneration in previously treated patients. J Ocul Pharmacol Ther.

[12] Cho H, Shah CP, Weber M, Heier JS (2013). Aflibercept for exudative AMD with persistent fluid on ranibizumab and/or bevacizumab. Br J Ophthalmol.

[13] Hariri A, Diniz B, Fou LV, Lam LA, Nittala MG, Sadda SR (2015). Quantitative OCT subanalysis of eyes with choroidal neovascularization switched from multiple injections of bevacizumab or ranibizumab to intravitreal aflibercept. Ophthalmic Surg Lasers Imaging Retina.

[14] Homer N, Grewal DS, Mirza RG, Lyon AT, Gill MK (2015). Transitioning to intravitreal aflibercept following a previous treat-and-extend dosing regimen in neovascular age-related macular degeneration: 24-month results. Eye (Lond).

[15] Moisseiev E, Katz G, Moisseiev J, Loewenstein A, Goldstein M, Lomnicky Y, Abend Y, Treister G, Goldenberg D, Levkovitch-Verbin H (2015). Switching treatment for neovascular age-related macular degeneration from bevacizumab to ranibizumab: who is likely to benefit from the switch?. Retina.

[16] Küçükerdönmez C, Gelisken F, Yoeruek E, Bartz-Schmidt KU, Leitritz MA (2015). Switching intravitreal anti-VEGF treatment in neovascular age-related macular degeneration. Eur J Ophthalmol.

[17] Heier JS, Brown DM, Chong V, Korobelnik JF, Kaiser PK, Nguyen QD (2012). Intravitreal aflibercept (VEGF trap-eye) in wet age-related macular degeneration. Ophthalmology.

[18] Seguin-Greenstein S, Lightman S, Tomkins-Netzer O (2016). A meta-analysis of studies evaluating visual and anatomical outcomes in patients with treatment resistant Neovascular age-related macular degeneration following switching to treatment with Aflibercept. J Ophthalmol.

